# Failure to Remove Bluetongue Serotype 8 Virus (BTV-8) From *in vitro* Produced and *in vivo* Derived Bovine Embryos and Subsequent Transmission of BTV-8 to Recipient Cows After Embryo Transfer

**DOI:** 10.3389/fvets.2019.00432

**Published:** 2019-12-05

**Authors:** Andy Haegeman, Leen Vandaele, Ilse De Leeuw, André P. Oliveira, Hans Nauwynck, Ann Van Soom, Kris De Clercq

**Affiliations:** ^1^Unit of Exotic and Particular Diseases, Sciensano, Brussels, Belgium; ^2^Department of Reproduction, Obstetrics and Herd Health, Ghent University, Merelbeke, Belgium; ^3^EPAMIG, Escola de Veterinaria da UFMG, Bolsista CAPES, Belo Horizonte, Brazil; ^4^Laboratory of Virology, Faculty of Veterinary Medicine, Ghent University, Merelbeke, Belgium

**Keywords:** bovine embryo, IETS guidelines, Bluetongue virus, BTV-8, transmission

## Abstract

The behavior of BTV-8 in cattle is different from most other serotypes not only with regards to clinical signs but certainly with respect to virus transmission (transplacental, contact). Therefore, the possibility of virus transmission by means of embryo transfer was examined by *in vitro* exposure of *in vitro* produced and *in vivo* derived bovine blastocysts to BTV-8 followed by different washing protocols, including longer exposure times (up to 120 s) to 0.25% trypsin at room temperature or at 37°C. None of the washing protocols used was successful in removing the viral genome completely from the *in vitro* produced and *in vivo* derived embryos as was demonstrated by real-time PCR. Moreover, BTV-8 virus was transmitted to recipient cows after embryo transfer of *in vivo* derived BTV8-exposed embryos, which had been subjected to routine decontamination as recommended by IETS, consisting of 5 washes in PBS followed by a double treatment of 0.25% trypsin for 45s at 37°C, and an additional 5 washes in PBS with 2% FCS. This study clearly demonstrates the necessity of vigorous application of the directives for screening of potential donors and the collected embryos, especially in regions with BTV-8, to prevent transmission of the disease.

## Introduction

Bluetongue virus (BTV) is a segmented double stranded RNA virus belonging to the genus Orbivirus, family Reoviridae (1) and is the causative agent for bluetongue disease. The disease has a significant impact on naïve populations and although BTV can infect all ruminant species, clinical signs are usually confined to sheep and white-tailed deer (2, 3). In epizootic situations the virus has the potential to cause severe socio-economic problems (4) due to loss of productivity, international movement restrictions, and lengthy and costly regulatory testing requirements of livestock and germ cell. The main transmission route for BTV is by biting midges (Culicoides spp.), but data have been published on contact transmission of BTV-8 (5) and BTV-26 (6). As human intervention in bovine reproduction has become common practice, with artificial insemination and embryo transfer being routinely used in cattle breeding, other possible transmission routes need to be considered. Shedding of BTV in semen is considered to be rare in ruminants (7) and only occurs during and/or directly after the viraemic period (8). This has been mainly observed for laboratory-adapted strains (BTV-1, BTV-23) but can also occur with wild type strains (BTV-23) (8). Although not completely elucidated, the presence of BTV in the seminal plasma of bulls is thought to be caused by the infiltration of infected blood cells due to injury or inflammation of the genital tract (8). The risk of transmitting BTV by embryo transfer is considered to be negligible by the International Embryo Transfer Society (IETS) when their guidelines for embryo washing/trypsin treatment are strictly followed (9, 10). This has been substantiated by experimental findings that when these guidelines are applied, *in vitro* or *in vivo* infection of the embryos does not result in the transmission of the virus to recipient cows (11–14) or ewes (15, 16) and their offspring. However, the emergence of BTV-8 in Central and Northern Europe in 2006–2009 (4, 17) did not only challenge our understanding of the geographic distribution of BTV and its potential vectors but numerous observations and experiments clearly demonstrated the atypical behavior of this particular serotype (18, 19). There was not only a significant increase in morbidity and mortality in cattle and offspring (20, 21) but infectious virus could readily be detected and isolated from bovine semen samples in the absence of contaminating blood cells (22). The fact that BTV-8 seems to interact differently with the genital tract compared to the other serotypes is also corroborated by other observations. Just as seminal shedding, transplacental infection was considered to be associated only with vaccine or laboratory-adapted BTV strains (23–25). However during the BTV-8 epizootic in Central and Northern Europe in 2006–2009 vertical transmission could be demonstrated on numerous occasions (26, 27). This potential of BTV-8 to be vertically transmitted resulted in increased numbers of abortions/stillborns and birth abnormalities and might be related to active virus replication as was shown in *in vitro* exposed bovine hatched embryos (28–30). The underlying genetic reason for the atypical behavior of BTV-8 still has to be clarified which makes it difficult to estimate the true extent of its different behavior. In view of the apparent altered interaction of BTV-8 with the reproductive system, it was the purpose of this study to examine the possibility of BTV-8 transmission by means of embryo transfer following different washing/trypsin protocols, including the one advocated by the IETS. Both *in vitro* produced and *in vivo* derived embryos were included in this study in alignment with current bovine assisted reproductive techniques.

## Materials and Methods

### Virus

The BTV-8 strain used (Bel2006/2) was isolated from an infected sheep during the 2006 epidemic through one passage on embryonated chicken eggs (ECE) and 5 passages on Baby Hamster Kidney (BHK-21) cells (ATCC-CCL10) as described by Toussaint et al. (17).

### Embryo Collection

#### *In vitro* Production of Bovine Blastocysts

Bovine blastocysts (*n* = 105) were produced by the following *in vitro* methods: after collecting bovine ovaries from an abattoir, the oocytes were aspirated from follicles measuring between 4 and 8 mm in diameter and cultured for 20–24 h at 38.5°C in 5% CO_2_ in air in groups of 100 in 500 μL modified bicarbonate buffered TCM-199 supplemented with 20% heat-treated fetal calf serum (FCS) (Biochrom AG, Berlin, Germany). Spermatozoa were separated from frozen-thawed bovine semen using Percoll-gradient centrifugation (Pharmacia, Uppsala, Sweden), and then washed. The mature oocytes were incubated with a sperm (sp) concentration of 1 × 10^6^ sp/mL in an *in vitro* fertilization medium consisting of bicarbonate buffered Tyrode albumin lactate pyruvate (TALP) solution, supplemented with bovine serum albumin (BSA, fraction V, A6003, Sigma-Aldrich, Bornem, Belgium) (6 mg/mL) and heparin (25 μg/mL). After 20–24 h of incubation the presumed zygotes were vortexed to remove excess sperm and cumulus cells and subsequently cultured for a further 7 days in 50 μL droplets of synthetic oviduct fluid supplemented with amino acids and FCS (SOFaa + 5% FCS) in an atmosphere of 5% CO_2_, 5% O_2_, and 90% N_2_ under mineral oil (Sigma-Aldrich).

#### In vivo Derived Embryos

Donor cows (*n* = 2) were synchronized and super-ovulated using Stimufol® (Ulg, Liége, Belgium) and subsequently inseminated. Donor cows (blood at the start of the synchronization) and bull (blood and sperm) tested negative in the BTV real-time RT-PCR (RT-qPCR) [see Virus isolation on embryonated chicken eggs (ECE)]. At 6.5 days post insemination (dpi), embryos (*n* = 14 and *n* = 3) were non-surgically collected by uterine flushing.

### Viral Exposure

At 7 days post insemination (dpi) for *in vitro* produced embryos and at 6.5 dpi for *in vivo* derived embryos, groups of 4 to 8 zona-intact embryos were placed in 800 μL of minimal essential medium (MEM), containing 10^4.9^ TCID50/ ml of BTV-8, a titer that can be found in semen from bulls naturally infected with BTV-8 (22), and incubated for 1 h at 39°C in 5% CO_2_ incubator (28). In total 98 *in vitro* produced embryos and 17 *in vivo* derived embryos were exposed to BTV8. Mock-exposure of 7 zona-intact blastocysts, *in vitro* produced, was performed in 800 μL SOF or 800 μM MEM without virus to evaluate any negative effects of MEM on blastocyst viability.

### Embryo Washing and Trypsin Treatment Procedures

#### Evaluation of Washing Procedures of BTV Exposed Embryos (*in vitro* Produced)

##### Preliminary evaluation of the decontamination of *in vitro* produced bovine embryos following the routine IETS procedure (experiment 1)

It was the purpose of this preliminary experiment to look at the efficacy of the routinely used IETS treatment/wash procedures to eliminate BTV8 from the *in vitro* produced bovine embryos. For this purpose 8 *in vitro* produced bovine embryos were exposed to BTV8 (as described in section Viral exposure) and dived in two groups. The first group was not washed/treated and functioned as a control group, while the bovine embryos in the second group were washed and treated as follows: the embryos were washed individually in 5 consecutive petri dishes containing PBS with gentamycin (50 mg/L) and 0.4% BSA, without Ca and Mg. Subsequently, the embryos were exposed to 2 consecutive 0.25% trypsin (Invitrogen, Carlsbad, CA, 25050-014) treatments by incubation for 45s in a 5% CO_2_ incubator at 37°C. Finally, another 5 consecutive washes in PBS with 2% FCS were performed. Each petri dish contained at least 2 mL of medium and was gently agitated between washes. Embryos were transferred in a maximum of 7 μL of medium and a new tip was used after every wash step. Washes 1–5 and washes 6–10 were pooled. The pooled washing fluids, trypsin liquid and the washed/treated embryos were stored at −80°C for real-time PCR evaluation.

##### Evaluation of increased duration of exposure of virus-exposed *in vitro* produced bovine embryos to trypsin at room temperature and at 37 °C (experiment 2)

For the second *in vitro* experiment, three different types of trypsin treatments (T45–T120) were evaluated at 2 different incubation temperatures, namely at 37°C (G37) and at room temperature (G20), resulting in six different treatment combinations (Table 1). Per treatment the experiment was carried out in triplicate whereby each sample (replicate) consisted of 5 embryos. Each sample (E) was washed five times in PBS without BSA followed by two treatments in 0.25% trypsin-EDTA for either 45s (T45), 60s (T60), or 120s (T120) and then followed by ten washes in PBS + 0.4% BSA. For each step, the embryos were transferred in a maximum of 7 μL of medium and a new tip was used after every wash step. Only washes 11 to 15 were pooled and are indicated as W11.

**Table 1:** Experimental set-up of the treatment procedure for the second *in vitro* experiment; E: Sample consisting of 5 embryos, Between brackets: the group assignment.

**Incubation time**	**At 37^**°**^C**	**At RT (20^**°**^C)**
**Wash and treatment procedures**		
**Wash step 1–5**		
**Trypsin treatment 1 and 2**		
45s	E1, E4, E7 (G37T45)	E10, E13, E16 (G20T45)
60s	E2, E5, E8 (G37T60)	E11, E14, E17 (G20T60)
120s	E3, E6, E9 (G37T120)	E12, E15, E18 (G20T120)

*E1 was excluded due to problems during the washing/trypsin treatment as explained in section Experiment 2: Evaluation of increased duration of exposure of virus-exposed in vitro produced bovine embryos to trypsin at room temperature and at 37°C*.

All washed/treated embryos, pools and individual wash/trypsin fluids were analyzed for the presence of BTV genome using RT-qPCR and virus isolation.

#### *In vitro* and *in vivo* Evaluation of the Routine IETS Wash/Treatment Procedure of BTV Exposed Embryos (*in vivo* Derived) (Experiment 3)

##### Washing and trypsin treatment

Embryos were either washed in pairs (*n* = 14; 7 pairs) or separately (*n* = 3) using identical washing and trypsin conditions as described for the embryos in the first *in vitro* experiment [see Preliminary evaluation of the decontamination of *in vitro* produced bovine embryos following the routine IETS procedure (Experiment 1)]. Washes 1–5 and washes 6–10, from the embryos washed in pairs, were pooled and analyzed for the presence of BTV-8 genome using RT-qPCR. Embryos which were not used for embryo transfer (see Embryo transfer) were similarly used for real-time PCR.

##### Embryo transfer

All the washed/treated *in vivo* derived embryos (*n* = 17) were examined for their suitability for embryo transfer in donor cows. In total 8 embryos, which had reached the morula or blastocyst stage were selected and washed in pairs. The embryos which were not selected for transfer, consisting of morulae, and degenerated or unfertilized oocytes, were washed following the same protocol (separately or in group). Three pairs of *in vivo* derived washed embryos were loaded in straws and transferred to three BTV negative recipient cows. The fourth pair was used for real-time PCR analysis. Two sentinel cows served as control. Cows were bled twice weekly and blood and serum samples were analyzed for the presence of BTV-8 RNA (RT-qPCR) and antibodies against BTV-8. The protocol was approved by the ethical committee of the faculty of Veterinary Medicine (Ghent, authorization number EC2011/094.

### Antibody ELISA

All sera were tested for the presence of BTV-specific antibodies by means of a commercially available competitive ELISA (c-ELISA) (ID Screen® Blue Tongue Competition, ID VET, Montpellier, France) performed according to the instructions provided by the manufacturer. Results were expressed as a percentage negativity (PN) compared to the negative kit control and were classified into positive (PN ≤ 65), doubtful (PN > 65 but ≤ 75), and negative (PN > 75) results based on the optimal cut-off point for diagnostic purposes of 65 PN determined by Vandenbussche et al. (31).

### RNA Extraction

RNA extractions were performed using the NucleoSpin® RNA Virus kit (Machery-Nagel, Düren, Germany) according to the manufacturer's recommendations with the exception of the addition of an external control (EC) to the RAV1 buffer (32). Hearts of chicken embryos were pre-treated as described in Garigliany et al. (33).

### Real-Time RT-PCR (RT-qPCR)

The efficiency of the different washing techniques and trypsin temperature for virus removal was evaluated by using a non-serotype specific quantitative reverse-transcription PCR assay targeting BTV segment 5 (pan-BTV/S5 RT-qPCR) according to the method described by Vandenbussche et al. (32) on embryos, washes and blood and organ samples. Test results were classified as follows: Crossing Point values (Cp-values) <40.0 were classified as positive, Cp-values ≥40.0 but <45.0 were classified as doubtful, and Cp-values ≥45.0 were classified as negative.

### Virus Isolation on Embryonated Chicken Eggs (ECE)

Virus isolations from washed/treated *in vitro* produced embryos, washing fluids and trypsin residues from Evaluation of washing procedures of BTV exposed embryos (*in vitro* produced). Two were performed as described for blood samples in Toussaint et al. (17) whereby 5 ECEs were used per sample. Passages on ECEs were done by collecting blood from chicken embryos that were still alive at 7dpi. This blood was 10 times diluted in PBS supplemented with 0.2% gentamycin and one hundred micro liter of this dilution was inoculated in new ECE. A sample was considered to be negative if all 5 ECEs were still viable after 7 dpi. Samples were considered to be positive if an ECE died between 2 and 7 dpi and the presence of BTV was confirmed by real-time PCR. The latter was achieved by homogenizing the heart of a dead embryo followed by RNA extraction and subsequent real-time PCR as described in 2.6 and 2.7, respectively.

## Results

### Experiment 1: Preliminary Evaluation of the Decontamination of *in vitro* Produced Bovine Embryos Following the Routine IETS Procedure

The four unwashed/untreated embryos of the control group were all positive for BTV with Cp-values between 31.2 and 33.9. When looking at the washed/treated group, all the pools of the first wash steps (1–5) were positive with Cp-values between 29.4 and 30. In contrast, the pools for wash step 6–10 were all negative. When the washed/treated embryos themselves were examined, two were found to be negative, one doubtful, and one was positive with a Cp value of 37.7.

### Experiment 2: Evaluation of Increased Duration of Exposure of Virus-Exposed *in vitro* Produced Bovine Embryos to Trypsin at Room Temperature and at 37°C

**Figure 1:**
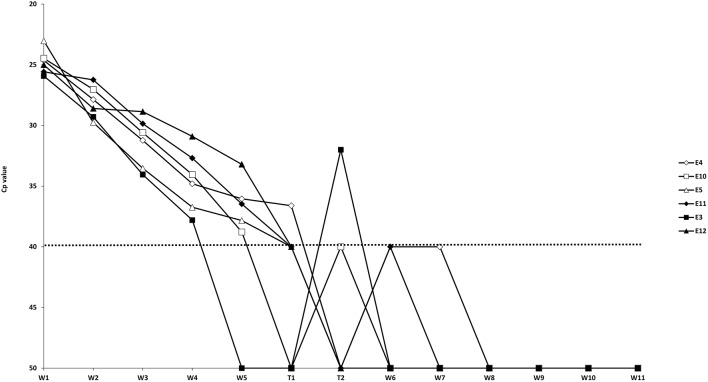
Initial RT-qPCR-results of the wash (W)/trypsin (T) fluids. The dotted line represents the cut-off for RT-qPCR positivity. E: Sample consisting of 5 embryos.

**Figure 2:**
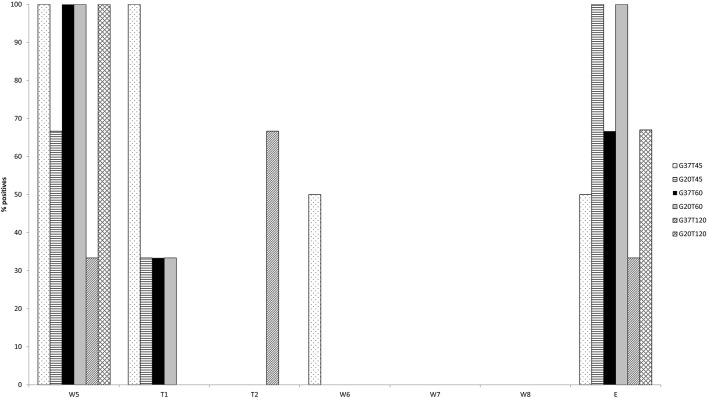
RT-qPCR results of the wash step (W) 5–8, including both trypsin treatments and the embryos (E) after all washing steps. The percentage of positives are represented per wash/trypsin treatment group (as defined in Table 1).

The results of the preliminary evaluation of the routinely used IETS wash/treatment procedure with pooled wash steps (Experiment 1) asked for a more in detail evaluation of individual wash and trypsin steps. In this second experiment the washing steps were analyzed individually and different trypsin treatments were evaluated (Table 1). Due to problems during the washing/trypsin treatment of the G37T45 group in the second *in vitro* experiment, one of the triplicate repeats was excluded (E1), meaning that G37T45 consisted only of 2 replicates (E4 and E7). The initial RT-qPCR screening of the wash and trypsin fluids containing one replicate of each of the six washing/temperatures combinations (E4, E10, E5, E11, E3, and E12) showed a decreasing viral load in each subsequent wash step (Figure 1). Five of the six samples were still positive at wash step five (W5), with Cp's between 33.2 and 38.8, while only one of the six remained positive after trypsin 45 (T45) and 60 (T60) seconds treatment. Additional doubtful results were obtained for T45 (*n* = 3) and T620 (*n* = 1). From wash step six (W6) onwards no positive results were obtained for all replicates although 2 and 1 sample were doubtful for, respectively W6 and W7. When analyzing the embryos after all the steps (washes and trypsin treatments), three replicates were found to be negative while 2 remained positive and one doubtful. Based on these results, W5–W8 of all the other replicates were tested as well as the remaining embryos. These results confirmed the initial screening with the majority of the samples being positive on W5 with rapidly decreasing Cp-values toward T45 and T60. Two differences were noted, however. One sample was positive in W6 and the majority of the embryos were positive, more precisely 12 of the 16 (Figure 2). One negative embryo was found in G37T45, G37T120, and G20T120 and one doubtful in G37T60. The average Cp-values of the washed embryos were very similar across the groups (average Cp-values between 35.5 and 36.2) with a small exception for G20T120 which had a slightly higher average Cp of 37.9. No positive RT-qPCR results were observed after W6 except one doubtful result for G37T45 at W7 from the initial screening. Although virus could be isolated on embryonated chicken eggs (ECE) from W1 and W3, no virus could be isolated from W5 onwards from the samples tested. Similarly, no virus was isolated from all the washed embryos even after 4 consecutive passages on ECE. The blood collected from the fourth passage remained negative on RT- PCR.

### Experiment 3: *In vitro* and *in vivo* Evaluation of the Routine IETS Wash/Treatment Procedure of BTV Exposed Embryos (*in vivo* Derived)

When looking at the real-time PCR results of the first pooled washing step (wash 1–5) of the embryos which were transferred to recipient cows, the Cp-value ranged from 28.9 to 29.5. Consistent with the data from experiment 1 and 2, the Cp-values of the second pool (wash 6–10) were a lot higher with 2 pools being borderline positive (38.9 and 39.5), 1 doubtful (Cp = 40) and 1 negative. As these embryos themselves could not be tested as they were transferred, an embryo pair which was not transferred but washed/treated was similarly analyzed by real-time PCR. Both individual embryos were positive with Cp-values of 32.3 and 32.5. When this is compared to the Cp values of the *in vitro* produced embryos under identical washing/treatment regime (G37T45), this was found to be slightly lower. The oocytes/embryos which were unsuited for embryo transfer were washed/treated and analyzed as well with real-time PCR. The Cp value that was obtained was very similar to those for the suited embryos: (1) the pools of the first wash steps were all positive (Cp-values of 27.7 to 32.2) while only one out of three was positive for the second pool (Cp 38.13); (2) the embryos themselves (*n* = 9) were all positive (Cp-values between 31.02 and 34.11) except one.

**Figure 3:**
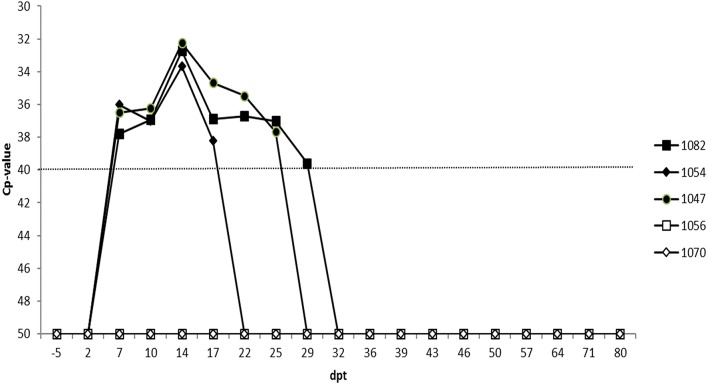
RT-qPCR blood results of the three recipient (1082, 1054, 1047) and 2 sentinel cows (1056, 1070). The dotted line represents the cut-off for RT-qPCR positivity. Dpt, days post transfer.

**Figure 4:**
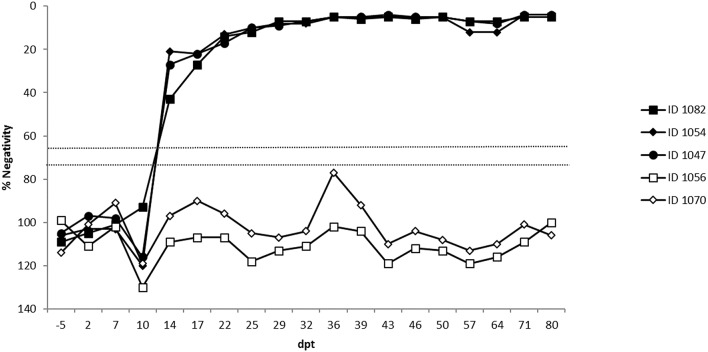
ELISA results of the three recipient (1082, 1054, 1047) and 2 sentinel cows (1056, 1070). Percentage negativity (PN): positive PN ≤ 65, doubtful PN > 65 but ≤ 75 and negative PN > 75. Dpt, days post transfer.

Two embryos were transferred to each of three recipient cows (identification number: 1047, 1052, and 1082). The latter two animals received embryos for which the second pool was negative while animal 1047 received a paired embryo sample for which the second pool scored doubtful. The two sentinel animals (identification number: 1056 and 1070) remained negative on ELISA and RT-qPCR for the complete duration of the experiment (i.e., 80 days post-transfer; dpt). The three recipient cows became viremic at the same time, namely 7 dpt and with similar Cp-values (Figure 3). Recipient 1047 and 1082 displayed a similar viremic profile and remained positive until 25 and 29 dpt, respectively. Cow 1052 had a shorter viremic period and was only positive until 17 dpt. All three recipient cows also displayed a very similar serological profile as they all seroconverted at 14 dpt and remained positive until the end of the experiment (Figure 4).

## Discussion

The risk of BTV transmission by embryo transfer has been considered to be negligible, when following the prescribed guidelines of the IETs. This is largely based upon animal experiments whereby BTV transmission to the recipient cows or ewes could not be demonstrated when the appropriate washing procedures were applied. These experiments were done using mainly BTV serotype 2, 4, 10, 11, 13, and 17 [reviewed by Wrathall et al. (10)]. Although the combined data spans several BTV serotypes, giving it more credibility, it needs to be mentioned that BTV has an important genomic diversity. This is reflected by the numerous serotypes which have been and are still being characterized (34). The serotype of BTV is defined by the structural protein VP2 whose coding sequence is the most variable of all the BTV segments. Inter-serotype diversity of VP2 can go as high as 59% on the nucleotide level and 73% on the deduced amino acid level (35). This protein is not only the most outer capsid protein (36) but is also implemented in cell attachment and entry (37). The combination of VP2's genomic variability and its function is a potential source of different virus serotype behavior. This is exemplified by BTV-8 which seems to interact differently with the components of the genital tract compared to the other wild type serotypes (vertical transmission, seminal shedding, contact transmission, …). Caution is therefore heeded regarding generalizations across serotypes and further investigations are warranted for serotypes displaying different behavior.

Correctly carrying out the washing procedure is an important step in the process of embryo transfer as BTV has great affinity for the zona pellucida of the embryo after *in vitro* exposure (38). This great affinity is clearly demonstrated in the Langston et al. study (39) where 12 consecutive wash steps failed to remove BTV from the bovine embryos as infectious virus could be recovered afterwards. Similar results were obtained in infected caprine and ovine embryos where 10 washes did not remove BTV completely (16, 40). Also in this study the affinity of BTV-8 for the embryos was noted as more than 80% of wash step 5 fluids were BTV-8 positive albeit with decreasing Cp-values. These data seems to be in contrast to the Venter et al. study (9) using ovine embryos where BTV (serotype 2 and 4) could only be detected in the first washing fluid and then even rarely. However, the used titers (1 × 10^2.88^ and 1 × 10^3.5^, respectively) in that study were lower than in our study. Even unwashed ova were not readily detected after a first passage on cell culture in the Venter study. The benefit of implementing trypsin treatments was clearly demonstrated as only 1 out of the 17 wash/treatment fluids remained positive in the first wash step following the treatments. This is supported by the finding of Ahmad et al. (41) where the washing fluids became negative after incubation with trypsin. In contrast to infected caprine embryos, where a double 0.25% trypsin treatment of 60s removed BTV-8 completely, none of the here evaluated trypsin treatments efficiently removed all traces of the BTV-8 genome from all the bovine embryos, not even the double 60s 0.25% trypsin treatment. Only small differences were seen between temperature groups (37°C group: 62.5% positives +12.5% doubtful vs. 88.9% positives in the room temperature group) and duration groups (positives: T45 80%; T60 83% + 16.7% doubtful; T120 66.7%). The small differences seen are probably due to more optimal conditions for trypsin (in regards to temperature and duration). Nevertheless, it is interesting to note that negative washing fluids did not prove that the embryos themselves were free from viral genome as was demonstrated by positive real-time PCR results. If there was still infectious virus present, however, could not be determined. Although the virus could not be isolated from the *in vitro* produced washed embryos, their Cp-values were high (> 35), meaning that the inability to isolate the virus could also be caused by a too low viral load. The instability of RNA needs also to be kept in mind specifically with the many wash and treatment steps that were a carried. The continued presence, therefore, of solely genomic RNA on the embryos seems unlikely. The importance of the inability to remove BTV-8 genome from the *in vitro* infected embryos is seen during the *in vivo* part of the study where the transfer of infected and washed, following the IETS guidelines, embryos resulted in the viremia and seroconversion in 100% of the recipients. The viremia seen in the recipient cows was in general shorter compared to naturally infected cattle (42). Although bluetongue viremia is generally perceived as prolonged (43) a short viremia (14 days) after infection with BTV-8 is no exception as shown by the extensive literature review by EFSA (44). The capacity to transmit the virus by embryo transfer to the recipients clearly demonstrates that infectious virus was present on the embryos after washing although it could not be isolated on ECE during the *in vitro* studies (Experiment 2). It needs to be stated that *in vivo* derived embryos were used for the *in vivo* part of the study while *in vitro* produced embryos were used for the *in vitro* part. This can be of importance as differences were seen in the ability to remove/inactivate BoHV-1 between *in vitro* produced and *in vivo* derived embryos using wash/trypsin treatments [reviewed by Wrathall et al. (10)]. Moreover, the zona pellucida of *in vivo* derived and *in vitro* produced bovine embryos is very different in its ability to bind virus (45). Although the washing steps were pooled for the *in vivo* trial (Experiment 3) instead of individually tested as in experiment 2, no differences were seen between both experiments in the PCR profiles and Cp-values of the washing fluids, trypsin liquids and the washed embryos. This indicated that the washing and trypsin treatments were equally ineffective in removing BTV-8 from *in vivo* derived and *in vitro* produced embryos. To our knowledge this is the first time that BTV-8 was transferred by means of embryo transfer when using the IETS guidelines for washing the embryos. Viremia and seroconversion in ewes was reported by Gilbert et al. (46) but the embryos used in this study were not washed or treated. In all other studies (9, 11, 14, 16) transmission was never reported if the IETS guidelines were followed. However, in these studies embryos were transferred from infected donors while in our study the embryos were exposed *in vitro* to the virus. The latter allows more control over the exposure of the embryo to the virus with parameters such as concentration of free virus, timeline and others. However, the question can be asked if the *in vitro* exposure is relevant for an *in vivo* situation. Firstly, the way of exposure of the embryos to the virus seems to be of importance, as more embryos had virus particles when they were exposed using an infected cell culture then when a viral suspension was used (38). Secondly, the question can be put forward if the BTV is able to come into contact with the embryo in order to infect or attach on it as harvested embryos from BTV infected donors are rarely reported to be positive (11). In many of the published studies the embryos are harvested at peak viremia in the blood under the hypothesis that this would be the time of the highest exposure of the embryos to the virus. However, data with regards to the organ distribution of BTV and its kinetics during viremia are not available to support this assumption. This could lead to a lesser or even unsuited time point for harvesting positive embryos. On the other hand, embryos can be exposed to BTV *in utero*, as the virus has been isolated from the uterus of cows infected with BTV11 (47). Furthermore, BTV circulates for a prolonged period of time in the blood of an infected cow, and the embryo can be exposed to the virus as a consequence of endometrial trauma during flushing when collecting the embryos (13). Although in most cases this was attributed to the infiltration of BTV positive red blood cells, free BTV was also found in cell free flush fluids. The latter would be capable of infecting the embryos, although it remains difficult uncertain to which virus titer bovine embryos are exposed to *in vivo*.

In summary, this study demonstrated that although extensive washing/trypsin treatment reduces and eliminates BTV-8 viral load from the washing fluids, it cannot completely clear the virus from bovine embryos spiked with BTV8. When the latter were transferred, it can result in virus transmission to the recipient.

## Data Availability Statement

All datasets generated for this study are included in the article/supplementary material.

## Ethics Statement

The animal study was reviewed and approved by the ethical committee of the faculty of Veterinary Medicine; Ghent University; authorization number EC2011/094.

## Author Contributions

AH was the author of the article and responsible of the real-time PCR evaluation of the samples Experiment 1 and 2. LV was responsible for Experiment 3. AO carried out the viral exposure, washing of the embryos in Experiment 1, 2. IL performed the real-time PCR and ELISA evaluations of Experiment 3. HN, AV, and KC were responsible for the cross–lab and cross institute coordination and critical scientific review of the data.

### Conflict of Interest

The authors declare that the research was conducted in the absence of any commercial or financial relationships that could be construed as a potential conflict of interest.
